# Thoracoscopic pulmonary metastasectomy in metastatic colorectal cancer: Surgical outcomes and prognostic factors

**DOI:** 10.1111/1759-7714.14132

**Published:** 2021-08-29

**Authors:** Ju Sik Yun, Eunchong Kim, Kook Joo Na, Sang Yun Song, In Seok Jeong, Sang Gi Oh

**Affiliations:** ^1^ Lung and Esophageal Cancer Clinic Chonnam National University Hwasun Hospital, Chonnam National University Medical School Jeollanamdo South Korea; ^2^ Department of Thoracic and Cardiovascular Surgery, Chonnam National University Hospital Chonnam National University Medical School Gwangju South Korea

**Keywords:** colorectal cancer, metastasectomy, thoracoscopy

## Abstract

**Background:**

This study aimed to confirm the effectiveness of thoracoscopic metastasectomy for colorectal cancer (CRC) and determine its prognostic factors.

**Methods:**

Of the 181 patients who underwent video‐assisted thoracoscopic surgery (VATS) for pulmonary metastases from CRC between 2011 and 2017, 173 were retrospectively reviewed. Surgical outcomes, long‐term survival, and the factors affecting the prognosis were analyzed.

**Results:**

The patients in the study were predominantly male (*n* = 104, 60.1%), and the median age was 65 years (range, 25–83 years). The median follow‐up time was 46 months (range, 0–114 months). The surgical procedures were 156 wedge resections, five segmentectomies, and 12 lobectomies. Conversion to thoracotomy was required in nine patients. The postoperative complication rate was 2.9%, and the in‐hospital mortality rate was 1.2%. The overall 1‐, 3‐, and 5‐year survival rates were 94.8%, 70.6%, and 51.8%, respectively. Univariate analysis showed that the prognostic factors for survival were age (*p* = 0.027), pathological stage of CRC (*p* = 0.019), prior extrathoracic metastasis (*p* = 0.005), preoperative carcinoembryonic antigen level (*p* = 0.020), number of pulmonary metastases (*p* = 0.011), and disease‐free interval (*p* = 0.026). In the multivariate analysis, two factors were related to prognosis: age (hazard ratio [HR], 1.881; 95% confidence interval [CI]; 1.189–2.976; *p* = 0.007) and prior extrathoracic metastasis (HR, 2.170; 95% CI; 1.269–3.711; *p* = 0.005).

**Conclusions:**

VATS for pulmonary metastasectomy for CRC can be performed relatively safely, and our results regarding long‐term survival are comparable with those of other studies. In this study, older age (≥70 years) and prior extrathoracic metastasis were independent prognostic factors of poor prognosis.

## Graphical abstract

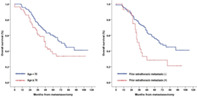

## INTRODUCTION

The lungs and liver are the most frequent sites of colorectal cancer (CRC) metastases. Surgical resection is considered optimal for local control, along with chemotherapy, and it has been widely practiced as a treatment for pulmonary CRC metastasis. Many studies have reported satisfactory long‐term survival rates and various prognostic factors of pulmonary metastasectomy for CRC, but surgical outcomes as well as the patients who would benefit from this procedure are still not clearly defined.^1^ To compensate for these shortcomings, several systematic reviews and meta‐analyses have been performed so far,^2^, ^3^, ^4^ and a randomized controlled trial in 93 patients has been published recently,^5^ but their interpretations still vary. Nonetheless, the National Comprehensive Cancer Network guidelines^6^ and expert consensus documents on pulmonary metastasectomy^7^ emphasize that this procedure should be considered in patients who meet the oncologic and medical criteria as determined by a multidisciplinary team.

As shown in the global trend, the number of patients with CRC is gradually increasing in Korea. In addition, with advancements in imaging technology such as computed tomography (CT), the number of patients considered for pulmonary metastasectomy has increased due to increased detection of pulmonary nodules suspected of metastasis.^8^ Considering the hematogenous dissemination of cancer, systemic chemotherapy is necessary, and since this technology is advancing as well, there may be disagreements about the role of surgical procedures. However, in our clinic, surgery is actively performed in patients who meet the criteria for pulmonary metastasectomy. We started performing video‐assisted thoracic surgery (VATS) in the early 2000s and have since been accumulating experience, which we have been actively utilizing in pulmonary metastasectomy since the late 2000s. The purpose of this study was to evaluate postoperative outcomes, recurrence, and long‐term survival rates in patients who underwent pulmonary metastasectomy for CRC via VATS to determine if they were comparable to the results from other studies. Furthermore, we aimed to analyze prognostic factors related to survival.

## METHODS

### Patients

This study was reviewed and approved by the Institutional Review Board of Chonnam National University Hwasun Hospital (approval no. CNUHH‐2021‐062), who waived the requirement for informed patient consent based due to the retrospective nature of the work.

The study included 181 consecutive patients who underwent pulmonary metastasectomy in our clinic for suspected lung metastasis after surgery for CRC and were pathologically diagnosed with metastasis between January 2011 and December 2017. Of these, eight patients were excluded from the analysis either because they underwent surgery for the purpose of histological diagnosis prior to chemotherapy due to multiple primary carcinomas, or because they underwent incomplete resection. When lung metastasis was suspected, the patient was referred to our clinic and was evaluated whether he/she satisfied the following oncological criteria for pulmonary metastasectomy^1^: the primary cancer must be controlled or controllable; there must be no uncontrolled or uncontrollable extrathoracic metastasis; all tumors must be resectable with adequate pulmonary reserve; and there are no alternative medical treatment options with lower morbidity rates.

After colorectal surgery, patients were followed up at 3‐month intervals for 2 years, then at 6‐month intervals for the next 3 years, and annually thereafter in our institution. Chest CT was performed at preoperative workup and semi‐annual follow‐up examinations.

Standard laboratory and pulmonary function tests were performed as routine preoperative tests. Thin‐slice (1.25 mm thickness) CT was performed 2–3 days before surgery, and metastatic nodule(s) were meticulously re‐evaluated.

Surgery was performed by three surgeons, and VATS was initially performed. Three thoracoports were usually used; no special localization technique was used for the nodule, and it was detected through intraoperative finger palpation or instrument sliding. In the case of severe pleural adhesion (six patients), or when the nodule was difficult to detect with finger palpation through a port incision (three patients), the operation was converted to open thoracotomy without hesitation. For the extent of resection, wedge resection was performed preferentially after considering the safety resection margin while saving the lung parenchyma, and segmentectomy or lobectomy was performed in cases where the location of the nodule was central or when there were multiple nodules. Lymph node sampling or dissection was only performed when metastasis was suspected in the examination of preoperative imaging, but not routinely. The length of hospital stay (in days), postoperative morbidity, and mortality rates were examined. To identify factors affecting survival rate, the following factors were analyzed: age; sex; CRC location, pathological stage, and histological differentiation; adjuvant chemotherapy after CRC resection; disease‐free interval between the colorectal resection and the first pulmonary resection; history of extrathoracic metastasis before pulmonary metastasectomy; preoperative carcinoembryonic antigen (CEA) level; maximal diameter and number of pulmonary metastasis; adjuvant chemotherapy after pulmonary metastasectomy; recurrence after pulmonary metastasectomy.

### Statistical analysis

Overall survival was calculated from the day of the first pulmonary metastasectomy to either the day of the last follow‐up or death. The Kaplan–Meier method was used for the analysis of all cumulative survival data, and log‐rank test was used for univariate analysis of categorical variables. Multivariate analysis was performed using the Cox proportional hazards regression model. Statistical significance was set at *p* < 0.05. Statistical analyses were performed using SPSS version 22 (IBM Corp.).

## RESULTS

The postoperative baseline characteristics of all 173 patients are shown in Table 1. The median age was 65 years, and the number of male patients was higher than that of female patients (60.1%). Regarding primary tumors, the rectum (58.4%) was affected more frequently than the colon. Most of the patients underwent laparoscopic surgery (86.7%) and adjuvant chemotherapy (86.1%). In patients with extrathoracic metastasis before metastasectomy, the liver was the most common site (25 patients), followed by the ovary (two patients) and brain (one patient). Of these, except for five patients who underwent radiofrequency ablation for liver metastasis, all patients had undergone curative surgery. According to the surgery‐related data (Table 2), conversion to thoracotomy occurred in nine patients, all of whom were in the first half of the study period. Postoperative complications occurred in five patients, of which three were mild, such as prolonged air leakage for more than one week, and two patients died in the hospital. Both were 75‐year‐old male patients with colon cancer who underwent pulmonary metastasectomy simultaneously with surgery for colon cancer. One patient died of acute respiratory distress syndrome due to pneumonia, and the other died from sudden cardiac arrest of unknown cause. Recurrence was observed in 65 patients during follow‐up. Among the extrathoracic recurrences (18 patients), the liver was the most common site (eight patients) followed by the pelvic cavity (five patients). Of these, except for three patients, 15 were administered adjuvant therapy. Thoracic recurrence occurred in the pleura (one patient), ribs (one patient), and lung parenchyma (45 patients), with the latter showing the highest frequency. Surgical resection for pleural nodules and radiotherapy for rib lesions were performed as adjuvant therapies. Most patients (*n* = 26) underwent repeat metastasectomy of the lungs; meanwhile, 15 patients underwent chemotherapy, and four patients refused chemotherapy and were only followed up closely.

**TABLE 1:** Preoperative baseline characteristics

Variable	Total no. of patients: 173
Age (years)	65 (Range, 25 ~ 83)
Gender, Male: female	104 (60.1):47
Colorectal cancer	
Colon: rectum	72 (41.6):101
Approach (laparoscopy: open laparotomy)^a^	150 (86.7):20
Pathological stage (I: II: III: IV)	8/30/80 (46.2)/55
Differentiation (Well: moderately:poorly)^a^	59: 85 (49.1):10
Adjuvant chemotherapy (Yes:No)	149 (86.1):22
History of extrathoracic metastasis (Yes:No)	28 (16.2):145
Disease‐free interval (months)	17 (range, 0–102)
Synchronous: 1–12: 13–24: 25–36: >36	32: 40: 48(27.7): 24:29

*Note*: Data are n (%) and median (range), unless otherwise indicated.

^a^ Data were missing for three (approach) and 19 (differentiation) patients.

**TABLE 2:** Surgical and postoperative data

Variable	Total no. of patients: 173
Preoperative CEA level (ng/ml)	
<5:≥5	129:44 (25.4)
Pulmonary metastasis	
Number (1:2:3:> 3)	111 (64.2):37:13:12
Location (right:left:bilateral)	77:65:31 (17.9)
Approach (VATS:conversion to thoracotomy)	164:9 (5.2)
Extent of resection (W:S:L)	156 (90.2):5: 12
Number (1:2:3:> 3)	111 (64.2):37:13:12
Length of hospital stay (days)	3 (Range, 1 ~ 15)
Postoperative complications	5 (2.9)
In‐hospital mortality	2 (1.2)
Recurrence after pulmonary metastasectomy	65 (37.6)
Thoracic	47 (27.2)
Extrathoracic	18 (10.4)

Abbreviations: L, lobectomy; S, segmentectomy; VATS, video‐assisted thoracoscopic surgery; W, wedge resection; Data are n (%) and median (range), unless otherwise indicated.

**FIGURE 1:**
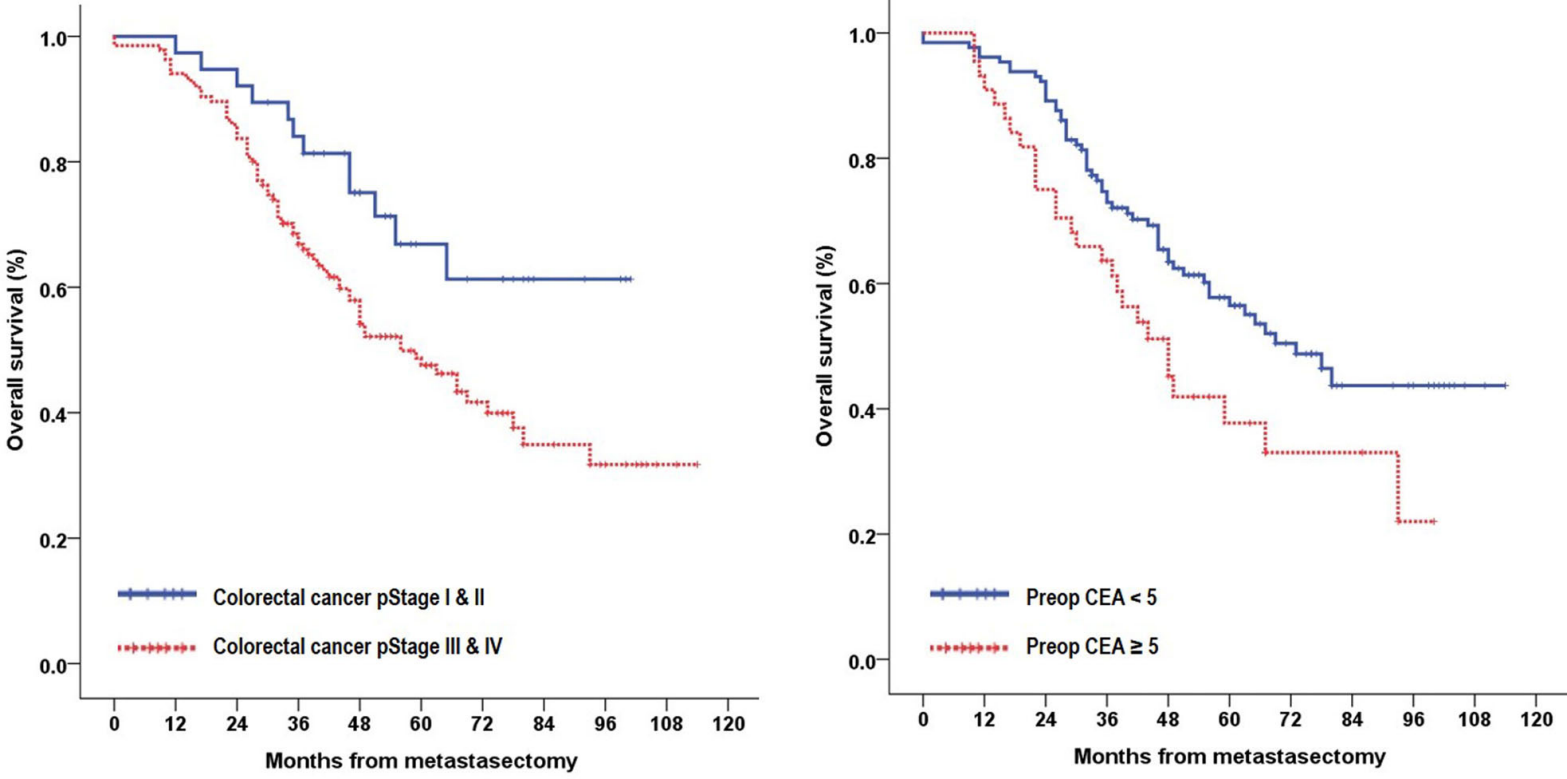
Kaplan–Meier survival curves for patients according to their pathological colorectal cancer stage (left) and preoperative serum CEA level (right). CEA, carcinoembryonic angigen

**FIGURE 2:**
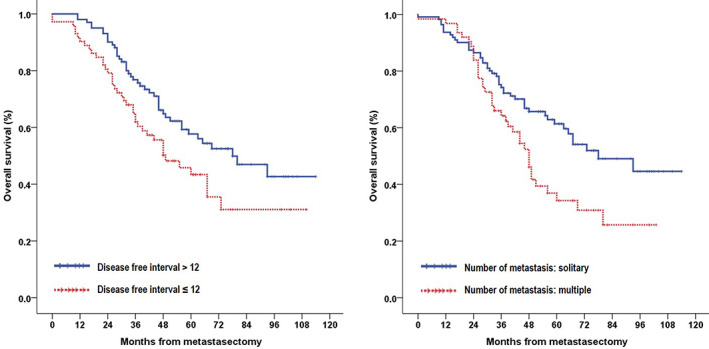
Kaplan–Meier survival curves for patients according to their disease‐free interval (left) and number of metastasis (right)

**FIGURE 3:**
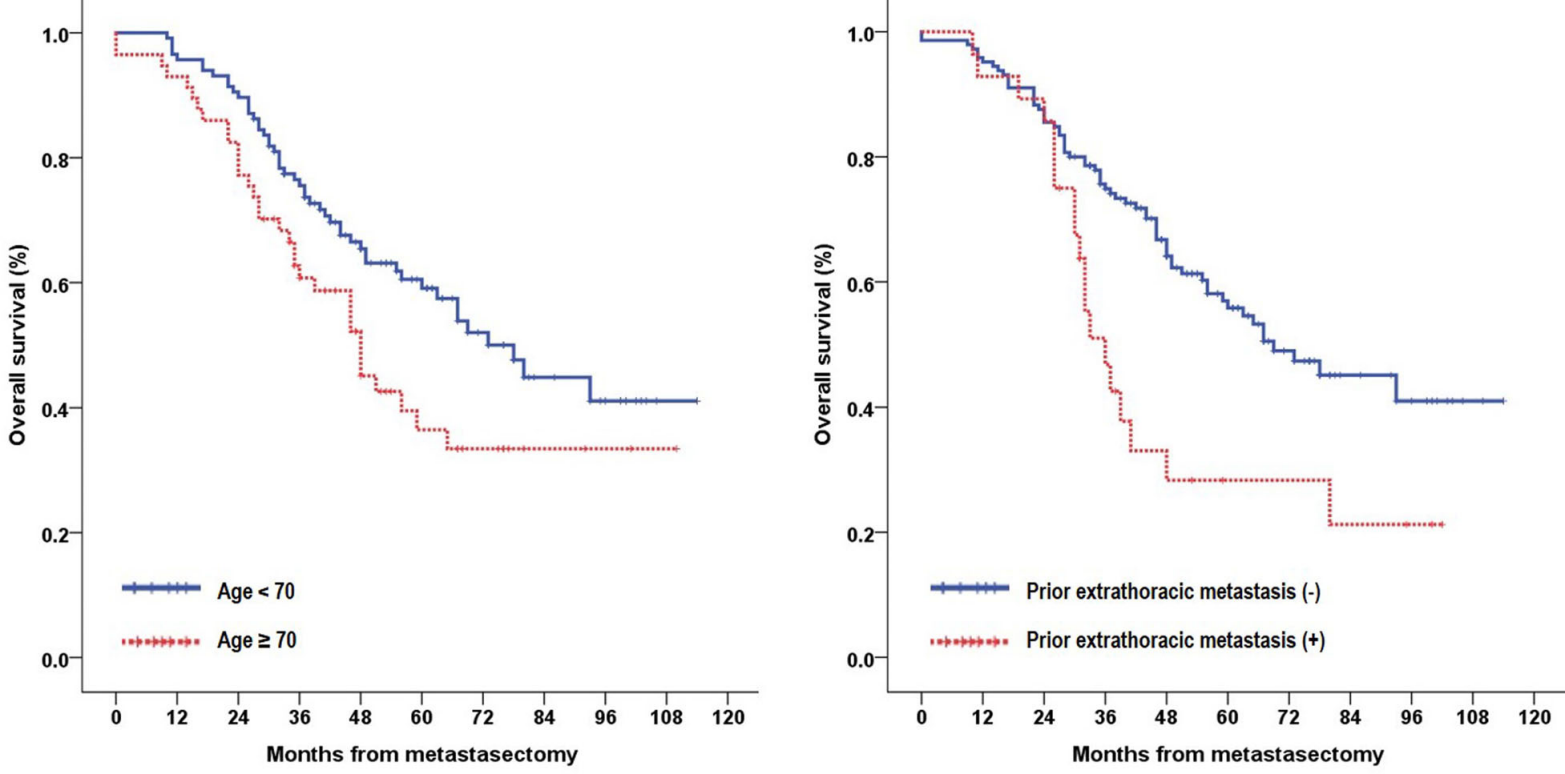
Kaplan–Meier survival curves for patients according to their age (left) and prior extrathoracic metastasis (right)

The median follow‐up period was 46 months (range, 0–114 months), and the overall 1‐, 3‐, and 5‐year survival rates were 94.8%, 70.6%, and 51.8%, respectively. In the univariate analysis (Table 3), age ≥ 70 years (*p* = 0.027), pathological stage of CRC (*p* = 0.019), disease‐free interval (*p* = 0.026), prior extrathoracic metastasis (*p* = 0.005), preoperative CEA level (*p* = 0.020), and number of metastases (*p* = 0.011) were prognostic factors that significantly influenced survival (Figures 1, 2, 3). In the multivariate analysis using factors that were significant in the univariate analysis, age (*p* = 0.007) and prior extrathoracic metastasis (*p* = 0.005) were identified as independent prognostic factors (Table 4). Comparison between the thoracic recurrence (*n* = 47) and control groups (*n* = 108) revealed that, except for those with confirmed extrathoracic metastasis after metastasectomy, there was a difference in the 5‐year survival rate (48.5% vs. 61.1%), but it was not statistically significant (*p* = 0.519).

**TABLE 3:** Overall survival rates and univariate analysis of prognostic factors

Variable	Value	No. of patients	5‐year survival	*p*‐value
Age (years)	<70	116	59.0%	0.027
	≥70	57	36.5%	
Sex	Male	104	50.5%	0.760
	Female	69	54.1%	
Location of primary tumor	Colon	52	53.8%	0.877
	Rectum	121	50.8%	
Pathological stage of CRC	1 & 2	38	66.7%	0.019
	3 & 4	135	47.8%	
Histological differentiation of CRC	Well	59	48.3%	0.865
	Moderate and poor	95	52.1%	
Adjuvant chemotherapy after CRC resection	(+)	149	51.7%	0.982
	(−)	22	51.4%	
Disease‐free interval (months)	≤12	72	43.6%	0.026
	>12	101	57.6%	
Prior extrathoracic metastasis	(+)	28	28.4%	0.005
	(−)	145	55.6%	
Preoperative CEA level (ng/mL)	<5	129	56.4%	0.020
	≥5	44	37.9%	
Maximum diameter of metastasis (cm)	<1	53	53.6%	0.560
	≥1	120	51.1%	
Number of metastasis	Solitary	111	61.4%	0.011
	Multiple	62	34.4%	
Adjuvant chemotherapy after pulmonary metastasectomy	(+)	146	50.5%	0.771
	(−)	27	57.7%	
Thoracic recurrence after pulmonary metastasectomy	(+)	47	48.5%	0.519
	(−)	108	61.1%	

Abbreviations: CEA, carcinoembryonic angigen; CRC, colorectal cancer.

**TABLE 4:** Multivariate analysis of prognostic factors for overall survival

Variable	HR	95% CI	*p*‐value
Age			
< 70	1		
≥ 70	1.881	1.189–2.976	0.007
Prior extrathoracic metastasis			
No	1		
Yes	2.170	1.269–3.711	0.005

Abbreviations: CI, confidence interval; HR, hazard ratio.

## DISCUSSION

To date, many studies have reported the survival rate and prognostic factors associated with pulmonary metastasectomy in CRC.^9^, ^10^, ^11^ According to the best evidence reported by Tsitsias et al., the median 5‐year survival rate was 52.5%, and the size and number of metastases, intrathoracic lymph node involvement, pre‐thoracotomy CEA levels, and response to induction chemotherapy were significant prognostic factors.^4^ In 2013, the Metastatic Lung Tumor Study Group of Japan analyzed a relatively large sample size of 1030 patients. The overall 5‐year survival rate was 53.5%, and tumor number and size, CEA level, lymph node involvement, and completeness of resection were reported as independent prognostic factors for survival.^1^ In addition, disease‐free interval, history of hepatic metastasis, and pathological stage (including T and/or N status) of CRC have been mentioned as prognostic factors in many reports, including meta‐analyses and review articles.^1^, ^2^, ^3^, ^12^, ^13^, ^14^ This study reported that age ≥70 years and prior extrathoracic metastasis were independent prognostic factors that indicated poor prognosis. Most of the existing literature rarely report age as a prognostic factor. Sponholz et al. claimed that morbidity, mortality, and overall survival after pulmonary metastasectomy with lymphadenectomy in older patients were comparable to those in younger patients.^15^ Generally, the older or elderly population is defined as those ≥65 years, but due to the severely increasing aging population, studies addressing surgical or oncological aspects are increasingly defining older age as ≥70 years.^16^ Chronological age alone cannot be said to reflect the patient's physiological status; however, functional and nutritional status, cognition, mobility, and social support inevitably deteriorate with aging. Taking these aspects into account, a comprehensive geriatric assessment prior to oncological surgery will help predict surgical outcomes,^17^ and this should not be an exception in the case of pulmonary metastasectomy. In this study, in‐hospital mortality occurred in two 75‐year‐old male patients who underwent pulmonary metastasectomy for colon cancer. In older patients >70 years, pulmonary metastasectomy with concomitant surgery for CRC should be carefully performed because the operation is relatively long and morbidity could be higher.

Because most studies to date are retrospective in nature and with small sample sizes, poor quality results were inevitably yielded; however, a randomized controlled trial of pulmonary metastasectomy in CRC was reported in May 2020.^5^ According to that report, survival in the metastasectomy group was comparable with that in many single‐arm follow‐up studies, and the survival rate of the control group (who did not undergo lung metastasectomy) was better than the assumed survival rate (overall estimated survival rates at 4 years were 47.1% in the control group and 44.4% in the metastasectomy group). When the same randomized participants were investigated for quality of life after some time, no benefit was reported for the metastasectomy patient group. However, when interpreting these data, one should consider the high survival rate of the control group, contrary to the small sample size and existing assumption that patients with lung metastases have a 5‐year overall survival rate <5%.^18^ Continuous sample size accumulation and large, well‐designed population studies will be required in the future. Iida et al. suggested that metastasis to the lung or other upstream organs can be regarded as a “semi‐local disease” when considering the hematogenous metastatic pathway of CRC.^1^ From this point of view, if complete resection is possible, pulmonary metastasectomy would be the preferred and reasonable treatment option.

The survival rate after pulmonary metastasectomy is improving with the development of surgical techniques and the accumulation of experience. Kim et al. stated that patients with previous or present liver metastases do not need to be excluded from pulmonary metastasectomy, and those with a history of hepatic metastasis can also expect surgical benefits if they have long disease‐free survival and a small number of lung metastases.^19^ In the case of recurrent pulmonary metastasis, there have been reports indicating that repeat pulmonary metastasectomy was safe and effective and showed satisfactory survival rates.^18^, ^20^ In this study, more than half of the patients with pulmonary recurrence (26/45, 57.8%) underwent repeat metastasectomy without any adverse effects. Although the comparison between the thoracic recurrence and control groups was not described in detail, there were no differences between the two groups in terms of clinical and surgical variables. In addition, in the survival rate analysis, there was a difference of approximately 12% in the 5‐year survival rate, but this difference was not statistically significant. However, even with recurrence, active treatment, including surgery, is expected to improve the survival rate.

VATS is already widely used globally and has various advantages in lung cancer, and its use is also gradually increasing in pulmonary metastasectomy. Moreover, it is not inferior to thoracotomy and has comparable survival rates.^21^, ^22^ However, VATS could be disadvantageous because localization of small nodules is more difficult, and it is easier to miss additional nodules that are not seen in CT images compared with thoracotomy. However, with the gradual improvement in preoperative localization techniques and the development of CT imaging technology, this aspect will no longer be considered a disadvantage. In this study, the median size of a metastatic pulmonary nodule was 1.2 cm and <1 cm in 53 patients. However, the surgeries were performed without special localization procedures by sufficiently experienced surgeons, and nodules were not detected in only three patients (1.7%), requiring a conversion to thoracotomy. Furthermore, in the case of repeat metastasectomy, all patients except for one were able to proceed without complications via VATS, including ipsilateral cases. Handy et al. reported that minimally invasive surgery, which has the advantage of shortened postoperative recovery and lessened effect on short‐term quality of life due to low morbidity, should be considered first in pulmonary metastasectomy.^7^

This study has several limitations. First, it was a retrospective study that targeted a small sample size in a single institution. Second, lymph node sampling or dissection was not performed routinely. However, only one case of intrathoracic lymph node metastasis was confirmed during the follow‐up period, and surgical resection was possible. Finally, the patients who were only closely followed up, or were administered adjuvant chemotherapy were included, which made it difficult to fully evaluate the benefits of repeat metastasectomy.

In conclusion, this study established that VATS pulmonary metastasectomy in CRC can be performed relatively safely with low morbidity and mortality and showed a comparable overall survival rate with that reported in other studies. In the univariate analysis, age, pathological stage of CRC, preoperative CEA level, prior extrathoracic metastasis, number of pulmonary metastases, and disease‐free interval were found to affect survival. The multivariate analysis confirmed that age and prior extrathoracic metastasis were independent prognostic factors. These factors may help identify the patient group that can benefit from metastasectomy.

## CONFLICT OF INTEREST

All authors have completed the ICMJE uniform disclosure form. The authors have no conflicts of interest to declare.
